# Reliability of ultra-thin metal films integrated onto embossed and bonded liquid-crystal-polymer (LCP) sheets for neural-interface applications

**DOI:** 10.3389/fnins.2026.1810147

**Published:** 2026-04-15

**Authors:** Ladan Jiracek, Ryan S. Wilkerson, Jack William Judy

**Affiliations:** Department of Electrical and Computer Engineering, University of Florida, Gainesville, FL, United States

**Keywords:** electrochemical impedance spectroscopy (EIS), flexible bioelectronics, implantable devices, liquid crystal polymer (LCP), neural interfaces, reactive accelerated aging (RAA), thin-film metallization

## Abstract

Liquid crystal polymer (LCP) is increasingly used in flexible implantable bioelectronic devices due to its low moisture uptake, chemical stability, and ability to form robust thermoplastic bonds. However, integrating fine-pitch thin-film metallization into bonded embossed LCP structures presents challenges related to pattern fidelity, bond integrity, alignment accuracy, and long-term electrical reliability, particularly when the metal thickness is small relative to the surface roughness. In this work, we present and characterize a fabrication process for integrating a 500-nm-thick sputtered Cr/Au thin-film metallization onto a 25-μm-thick embossed high-temperature LCP (HT-LCP) substrate, patterned into long (20 cm) and narrow (8 μm) traces using lift-off. Bond integrity between the metallized HT-LCP and a low-temperature LCP (LT-LCP) layer was evaluated using peel testing, while structural and electrical integrity were assessed using NanoCT imaging and resistance measurements. Long-term reliability was evaluated using reactive accelerated aging (RAA) at 87 °C in physiological saline with 10 mM hydrogen peroxide. The results show that the thin metal layer does not degrade bond strength and that embedded traces maintain structural and electrical integrity through bonding and aging. After 12 days of RAA testing, no measurable changes in electrical performance were observed. Electrochemical impedance spectroscopy demonstrated that electrodes coated with a 100-nm sputtered Pt layer exhibited approximately 2 × lower impedance than flat Pt electrodes, attributed to increased surface roughness. Additionally, the bonded LCP structure was thinned from 50 μm to 10 μm using CF4/O2 reactive ion etching with >90% uniformity. These results demonstrate that thin-film metallization integrated into bonded embossed LCP systems can achieve high interconnect density without compromising mechanical or electrical reliability. This work provides practical guidelines for the design of thin, flexible, and durable LCP-based implantable bioelectronic devices.

## Introduction

Miniaturized flexible implantable neuroelectronic devices require materials and fabrication processes that can achieve high-density metallization while maintaining long-term physical and electronic reliability in the body. Although conventional flexible substrates like polyimide have enabled the integration of thin-film microelectrode arrays, devices made with these materials use a layer-by-layer microfabrication process that has limited long-term reliability (Kuliasha and Judy, 2018; Cvančara et al., 2020; Jiracek-Sapieha et al., 2023; Fluker and Judy, 2025). Liquid Crystal Polymer (LCP) has emerged as a promising alternative dielectric material due to its extremely low moisture absorption (~0.1–0.4%), excellent chemical stability, and thermoplastic nature (Collyer, 1993; Lee et al., 2011; Jeong et al., 2013, 2016; Gwon et al., 2016a; Bihler et al., 2018; Woods et al., 2018; Ahn et al., 2019; Jeong et al., 2019). Since LCP is a thermoplastic, it can be heat-laminated in a process that merges multilayer structures by simply stacking the layers and pressing them together at elevated temperatures and pressures for a sufficient dwell time.

However, the chemical inertness of LCP, which is a beneficial trait for encapsulation, can complicate the strong attachment of thin metal layers onto its surface via chemical bonding (Jeong et al., 2013; Ahn et al., 2019; Jeong et al., 2019). To overcome this challenge, thin metal sheets are often attached to LCP by thermal compression bonding after the surface of the metal is roughened by an electroplating process that forms micron-scale features protruding ~2 μm from the surface (Culbertson, 1995; Chen et al., 2002; Jia et al., 2021). The thermoplastic nature of LCP, which causes it to flow into and around the protrusions on the roughened metal layer, creates a mechanically interlocked bond between the plated metal foil and the LCP. When the bonded metal layer is etched to form circuits, the LCP regions without metal have an embossed surface that is the negative image of the roughened metal foil that was removed. Subsequent bonding of a LCP layer with a lower melting temperature (LT-LCP) to the metallized LCP with a higher melting point (HT-LCP), causes the LT-LCP to flow into the embossed surface of the HT-LCP and again form a strong mechanically interlocked bond (Jeong et al., 2016).

**Figure 1:**
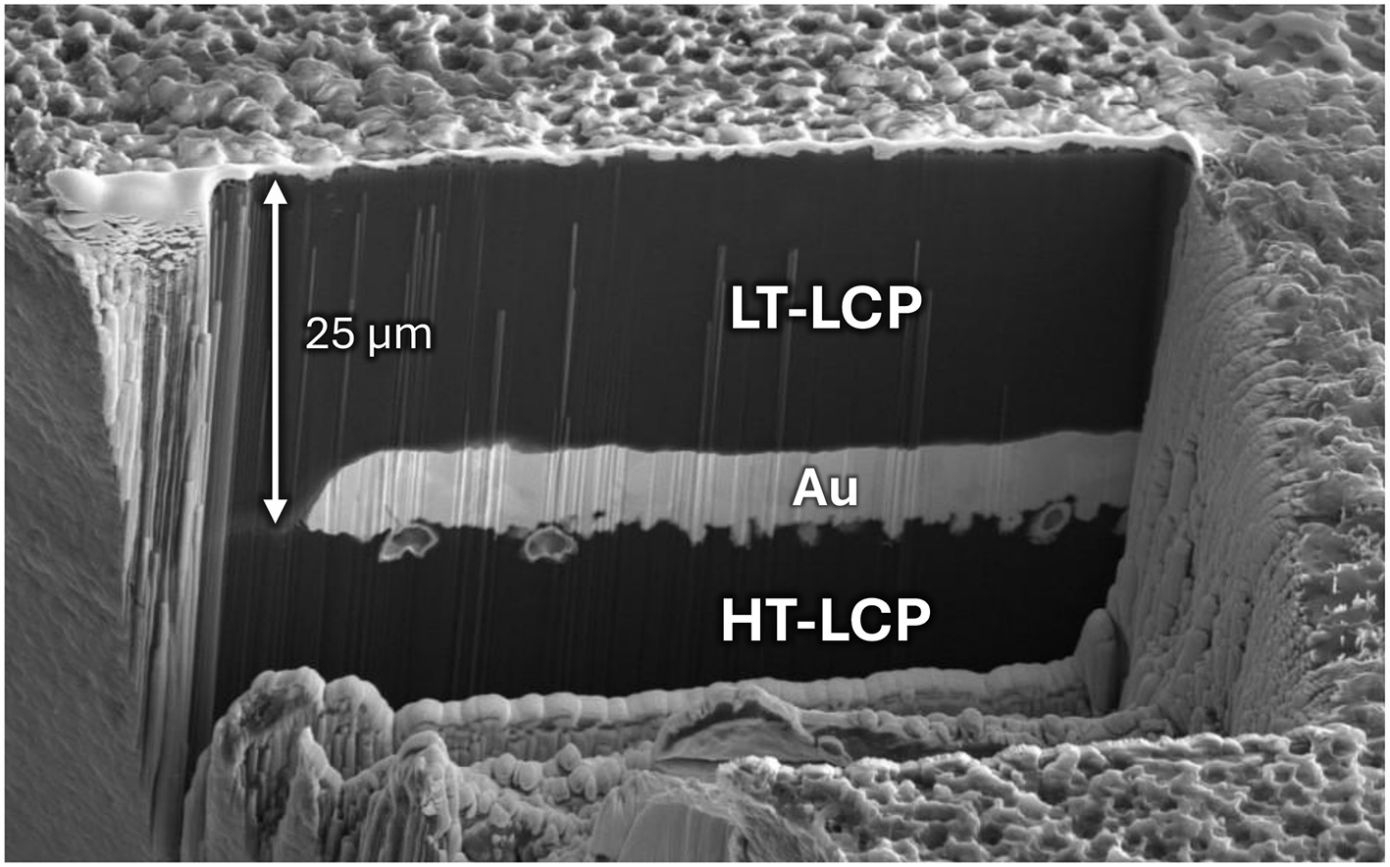
Focused-ion-beam SEM image of the cross section of a 25-μm-thick embossed LCP, covered with 5-μm-thick plated metal layer, that is bonded to a 25-μm-thick LCP layer. The bottom of the metal layer is mechanically interlocked with the top of the embossed LCP layer but the top of the metal layer is not interlocked with the bottom of the upper LCP layer.

**Figure 2:**
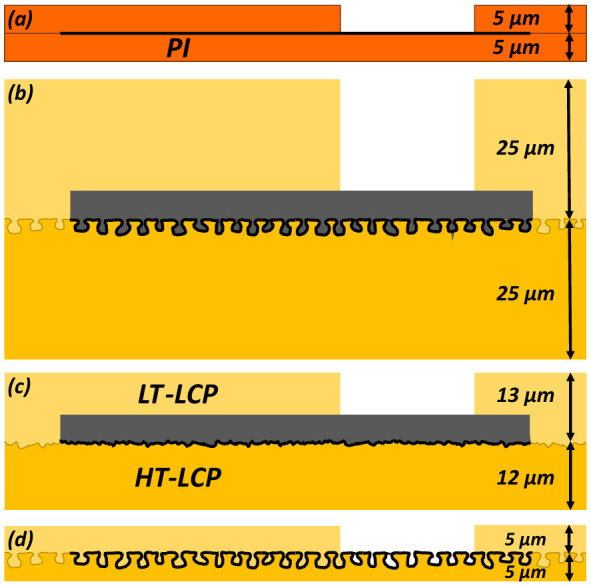
Motivation for thin-film metallization in ultra-thin LCP-based bioelectronic devices. Schematic comparison of common thick-metal flexible circuit approaches **(a)** vs. the thin-film LCP metallization strategy pursued in this work **(d)**. Conventional implementations often use metal layers thicker than several microns, which can dominate the overall device thickness and prevent ultra-thin (< 10 μm) implant architectures **(b)**. Thick metallization may also reduce polymer conformability over the metal features, resulting in limited mechanical interlocking at the top polymer-metal interface even when bottom interlocking is preserved **(c)**.

Dyconex AG produces neuroelectronic implants with a process that starts by removing all of the Cu metal foil bonded to an LCP sheet and then integrates a biocompatible microfabricated metal layer on top of the now embossed HT-LCP. This is done by first conformally depositing a thin palladium seed layer and then electroplating a thicker gold layer (~5 μm) on top of it. Since the thickness of the plated gold is more than twice the amplitude of the roughness of the embossed HT-LCP layer, it strongly attaches to the HT-LCP through mechanically interlocking but its smooth upper surface does not bond well to the LCP above it (Figures 1, 2b). However, since the upper LCP layer interlocks with the lower embossed LCP on all sides around patterned metal features, it effectively stays attached in close proximity to all sides of the plated metal (Bihler et al., 2018). Neuroelectronic devices made with this method have been shown to have excellent long-term reliability (Woods et al., 2018).

The Kim lab at Seoul National University has taken a different approach to metallize and form bonded LCP-based neuroelectronic implants. They used smoother un-embossed sheets of LCP, a thin sputtered seed layer (Ti: 50 nm/Au: 150 nm) layer, a thick electroplated gold layer (>5 μm) for the circuits, pads, and electrodes, then removed the seed layer. Since the surface of the HT-LCP is not embossed but rather has the much smaller-scale surface roughness that results from the extrusion process used to create the sheet (Figure 1c), chemical bonding is expected to play a more prominent role than bonding via mechanical interlocking. Indeed, the Kim lab has reported that the interface between the LT-LCP and the top of the metal layer is the interface where water ingress occurs most commonly and results in device failure after only 2–3 months in saline at 67 °C (Jeong et al., 2019). To address this problem, the Kim lab developed a mechanical interlocking approach that uses a photolithography-based approach to patterned narrow metal features around the perimeter of electrodes to interlock with the LT-LCP layer (Gwon et al., 2016b). They were able to improve the longevity of their devices by 4X to ~150 days when tested in saline at 75 °C. Since our approach uses surface texturing instead of additional layout features, it is much more space efficient.

To improve the performance and longevity of neuroelectronic interfaces, it is important to minimize foreign-body tissue responses that lead to the buildup of scar tissue and the loss of adjacent neurons that reduce the amplitude of recording neural signals and the generation of reactive oxygen species that can damage the implant. One method used to accomplish this goal is reducing the width and thickness (i.e., the cross-sectional area) of implants (Xie et al., 2015; Luan et al., 2017). Although LCP-metal-LCP bonding can result in very robust flexible circuits with relatively thin geometries when compared to conventional circuit boards, it has limitations that are problematic for neural-interface applications. One key limitation is that since the minimum thickness of commercially available LCP sheets is 25 μm, the minimum thickness of an LCP-metal-LCP neural interface is >50 μm (Figure 2b), which is 5–10 times thicker and thus 125 to 1,000 times more stiff than thin-film neuroelectronic interfaces (5–10 μm thick) made with polyimide or parylene (Figure 2a). This increased stiffness can contribute to larger foreign-body responses and reduced long-term neural recording performance. Although Dyconex AG does not thin their LCP-based neural implants, researchers at Seoul National University have used laser ablation to thin 50-μm-thick bonded LCP devices to 25 μm (Figure 2c). There is a need to develop processes for thinning LCP-based devices further to support greater mechanical flexibility, improved tissue integration, and increased functional longevity.

Integrating a thinner metal layer can help reduce the thickness and stiffness of the LCP-based implant. If the metal layer is significantly thinner than the roughness of the embossed surface, it should bond much more strongly to the LT-LCP layer than approaches with a smooth metal surface. This thin-metal approach may also reduce the likelihood of polymer-metal delamination by allowing the encapsulating LCP layer to conform more closely to the metal topography and maintain stronger mechanical interlocking. However, integrating very thin and fine-featured metal traces onto the rough surface of embossed LCP presents unique fabrication and reliability challenges (Dean et al., 2004, 2008). Here we report on work that uses a 0.5-μm-thick metal layer that is ~4 times thinner than the 2-μm-high roughness of the embossed surface (Figure 2d). We expect that the metal layer will bond to the LT-LCP about as strongly as the HT-LCP layer does. We also expect that it will challenge the reliable formation of long and narrow metal traces on it. Another challenge we address is the low optical transparency of LCP, which makes optical alignment through it to the buried metal layers difficult. The final challenge we report on is a process for uniformly thinning the LCP to a greater extent than previously reported.

## Materials and methods

### LCP materials

The devices tested were fabricated using 25-μm-thick embossed high-temperature liquid crystal polymer (HT-LCP) sheets supplied by Dyconex (Bassersdorf, Switzerland), which have a melting temperature *T*_*m*_ of 315 °C, as the substrate layer and 25-μm-thick low-temperature LCP (LT-LCP) also supplied by Dyconex, which have a *T*_*m*_ of 280 °C, as a lamination layer for encapsulation. This combination of HT-LCP and LT-LCP leverages the thermoplastic nature of LCP to enable LCP-metal-LCP sandwich structures, where the top LT-LCP layer can bond to the lower HT-LCP layer without the HT-LCP layer melting as well and causing the distortion of thin-film metal traces that are integrated onto it (Bihler et al., 2018). Both HT-LCP and LT-LCP strongly resist the absorption of moisture (< 0.5% at saturation).

### LCP-cleaning process

Since surface contamination can inhibit strong bonding between the LT-LCP and HT-LCP sheets by preventing intimate contact between the sheets and disrupting polymer interdiffusion at the interface, all LCP sheets were subjected to a cleaning protocol we established prior to bonding and metallization. This protocol consisted of the following steps: trisodium phosphate (TSP) sonication for 1 min, acetone sonication for 1 min, isopropanol sonication for 1 min, and deionized water sonication for 1 min, followed by drying with a nitrogen air gun. This approach was selected to remove handling residues (e.g., oils, dust, and adhesive contamination) while avoiding abrasive surface damage.

### Metallization process

**Figure 3:**
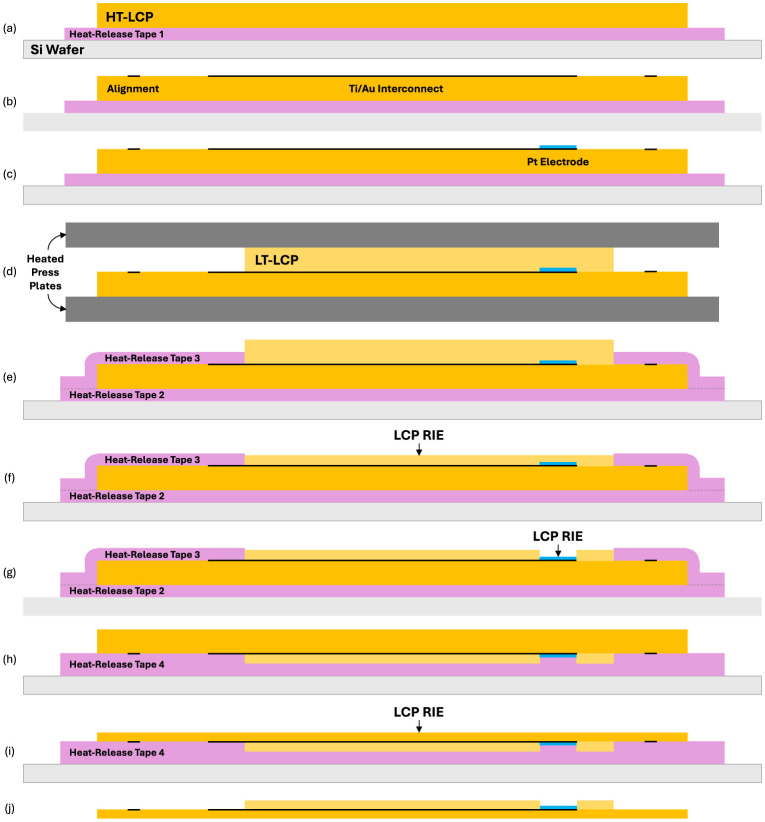
Schematic illustration of the fabrication process and multi-step alignment strategy used to produce the test samples **(a–f)**. Device shown in **(g)** has been flipped over in **(h)** and **(i)** and then flipped back in **(j)**.

As shown in Figure 3, the fabrication process begins with laminating the HT-LCP coupons onto silicon carrier wafers using a heat-release tape (Revalpha RA-95HS(N), Nitto Denko, Osaka, Japan) to provide a flat, mechanically stable, and easily handled substrate during processing (Figure 3a). A negative lift-off resist (AZ^®^ nLOF 2035, MicroChemicals GmbH) was spin-coated at 4,000 rpm for 60 s to obtain a resist thickness of ~2.5 μm (Figure 3d). This resist thickness was chosen because it can consistently cover the surface roughness (~2–3 μm) of the embossed HT-LCP sheets. A resist-to-metal thickness ratio of roughly 5:1 was sufficient for reliable lift off. After a soft bake is used to drive off excess solvent from the photoresist, contact lithography is used to expose the resist. The thin metal film consisted of a 5-nm-thick adhesion layer and a 500-nm-thick layer of Au. We used Cr as the adhesion layer in this work because we could reliably pattern and retain slightly narrower metal traces when using it instead of Ti. However, in future work Ti can be used as the adhesion layer to improve biocompatibility. For the devices characterized with electrochemical impedance spectroscopy, we formed electrodes out of a solid (i.e., not nanoporous) 100-nm-thick layer of platinum. All metal layers were deposited using a multi-source sputter deposition tool (CMS-18, Kurt J. Lesker) (Jefferson Hills, PA, United States) with 5 mtorr of Ar. Lift off was then performed using N-methyl-2-pyrrolidone (NMP) and mild agitation to remove the resist and regions of unwanted metal film.

### LCP bonding process

After the HT-LCP coupon was adhered to a carrier wafer with heat-release tape and the adhesion and Au metal layers were patterned using conventional lithography and lift off. The heat-release tape was then removed and a LT-LCP coupon was aligned and bonded to the larger metallized HT-LCP coupon. The thermal bonding process used a custom-designed lab press and bonding parameters previously determined to yield the best bond outcomes for the LCP sheets (temperature of 285 °C, pressure of 0.25 MPa, dwell time of 12 min, and controlled cooling) (Figure 3h). Following the dwell period, the heating elements were turned off and the stack was allowed to cool under pressure until the temperature decreased to approximately 250 °C. At that point, a slotted aluminum cooling fin was placed in contact with the heated press block (with thermal paste used to improve thermal contact), which accelerated heat removal while maintaining pressure on the bonded stack. This approach reduced the cooling time from approximately 30 min under passive ambient cooling to approximately 10 min. The bonding process causes the top LT-LCP to soften and flow, encapsulating the metal traces and fusing to the rough surface of the embossed HT-LCP. All bonded samples were inspected under a microscope to ensure there were no obvious defects, such as large unbonded bubbles or voids. Heat-release tape was then reapplied to facilitate further fabrication processes.

### LCP thinning procedure

In parallel with the bonding and metallization work, we evaluated a reactive-ion-etching (RIE) process to thin LCP substrates after metallization while preserving fine metal features. Thinning is necessary because the thinnest commercially available LCP films are approximately 25 μm thick, which results in a minimum LCP-based-interface thickness (i.e., LCP-metal-LCP stack) of ~50 μm. This is at least an order of magnitude thicker than many thin-film polymer-metal neural interfaces fabricated using polyimide or parylene. The desire to produce LCP-based devices of equivalent flexibility motivated the efforts to thin the LCP layers.

Thinning experiments were performed in a parallel-plate RIE system (790 RIE, Unaxis Inc, USA) using an oxygen-based plasma, with and without CF_4_, operated at 500 W. Rather than performing a formal design-of-experiments study, a limited set of etch-rate measurements was conducted on both LT-LCP and HT-LCP films to assess achievable etch rates, uniformity, and pattern fidelity. The plasma-chamber pressure (100 and 1,000 mTorr) and gas composition (O_2_ only vs. O_2_ and CF_4_) were varied to explore practical trade-offs between etch speed and uniformity.

### Alignment process

To produce a multilayer LCP-metal-LCP bioelectronic device that consists of exposed metal pads and electrodes and buried metal traces, precise alignment of etch holes through the top LT-LCP layer to the geometry of the buried metal layer is required. However, this alignment process is extremely challenging when using conventional optical lithography because LCP films are opaque over the visible wavelengths of light and only slightly translucent to infrared light. Since we did not have access to lithography equipment with x-ray alignment capability, we overcame this alignment challenge by adopting a multi-step strategy that used metal alignment marks located on a region of the larger HT-LCP coupon that is outside the area bonded to the LT-LCP and remain directly (Figure 3).

### Test structures and measurements

We designed several test structures to evaluate the quality and durability of the deposition and patterning of the thin Au metal layer on embossed LCP and within bonded LCP-metal-LCP laminates. Electrical continuity and resistance measurements were performed using microprobe tips connected to a handheld digital multimeter:

- **Resolution Test Structures:**

These included small arrays of sets of lines and spaces with equal widths and gaps that varied in size from 4 μm to 20 μm in steps of 2 μm (i.e., 4, 6, 8, 10, 12, 15, 20, 25, 30 μm). These devices, which were used to confirm the minimum achievable metal line width and gap, were examined by optical microscopy, NanoCT, and scanning electron microscopy (SEM).

- **Serpentine Electrical-Continuity Test Structures:**

To quantify the reliability to pattern long narrow thin-film metal lines on embossed LCP, we used 200-mm-long serpentine metal traces of varying line width (i.e., 4, 6, 8, 10, 12, 15, 20, 25, 30 μm). All serpentine test structures had a large gap (20 μm) between the in-plane folds of the serpentine to ensure there was no electrical shorting within the devices tested. The large spacing between adjacent segments was intentionally chosen so that failures would primarily reflect breaks in the narrow metal traces themselves rather than electrical shorting between neighboring traces.

- **Interdigitated Electrode (IDE) Test Structures:**

To quantify the reliability of maintaining electrical isolation between adjacent metal lines, we use pairs of 20-mm-long interdigitated metal combs that each had a different gap between the metal lines (i.e., 4, 6, 8, 10, 12, 15, 20, 25, 30 μm). The width of the metal lines was large enough (50 μm) to prevent open circuits within the devices tested. This complementary structure was designed so that the metal lines themselves would remain electrically robust while the gap spacing determined whether electrical isolation between neighboring traces was maintained.

- **Metallized Bond-Strength Samples:**

To quantify the impact of the thin metal layer on bond strength, we coated embossed HT-LCP coupons with a 500-nm-thick layer of sputtered metal. Prior to bonding an identically sized LT-LCP coupon to it, a 10-μm-thick polyimide coupon was used to prevent one end of the metallized HT-LCP coupon from bonding to the LT-LCP coupon. Pull tests were performed using a texture analyzer (TA.XT, Texture Technologies Corp.) to measure the strength of the bond. Normalized linear bond strength was calculated from the measured peel force by dividing it by the width of the bond. The results were compared to control LCP-LCP coupons bonded with the same parameters but without the thin metal layer.

- **Bonded Serpentine Electrical-Continuity Test Structures:**

We fabricated large and long serpentine test structures that could quantify the reliability of forming long and narrow metal traces integrated into a bonded LCP-metal-LCP stack. To do this without requiring alignment and etching through the top LCP layer, we integrated a series of long tap lines that connected to different positions along the length of each serpentine to large metal pads positioned outside of the bonded area. Again, the width of the serpentine was varied (i.e., 4, 6, 8, 10, 12, 15, 20, 25, 30 μm). These devices allowed us to assess continuity as a function of bonded trace width and length. Measurements were performed both before LCP bonding and after LCP bonding. Segments that yielded resistances of 100 to 2,000 Ω were categorized as being good (i.e., expected/acceptable). Other segments that yielded significantly higher resistances and indeed open circuits, which could be due to failures during lift off or geometrical distortions and stresses caused by the bonding process, were deemed to be failures.

- **Exposed Electrodes:**

To test the performance of electrodes for bioelectronic stimulation and recording, devices were produced that had etch holes through the top LT-LCP layer that were aligned to and revealed the surface of sputtered metal electrodes. The electrodes were made from a solid (i.e., not nanoporous) 100-nm-thick layer of sputtered Pt. The diameter of the circular electrode sites varied from 50 to 400 μm. All electrodes were connected by buried metal traces to exposed bond pads on the opposite end of the device.

### Electrochemical impedance spectroscopy (EIS)

We characterized the impedance of the exposed electrodes using EIS in phosphate-buffered saline (PBS) to simulate body fluid. A three-electrode setup was used with macroscopic Ag/AgCl reference and Pt counter electrodes. Impedance spectra were recorded from 100,000 to 0.1 Hz with a 200 mV RMS sinusoid at open-circuit potential. We chose to use a sinusoid with a large amplitude to improve the signal-to-noise ratio at low frequencies while remaining within the practical electrochemical stability window of noble-metal electrodes in aqueous electrolyte. Although smaller perturbation amplitudes are commonly used to ensure operation strictly within the linear response regime, we selected 200 mV RMS because it provided significantly improved signal-to-noise at low frequencies where the electrode impedance is highest. We note that the larger excitation amplitude could introduce minor non-linear contributions to the impedance response, particularly at low frequencies, and therefore the results should be interpreted primarily in a comparative sense across electrode geometries rather than as a precise electrochemical model of the interface. This allowed us to assess the impact of surface roughness and metal deposition method on electrode impedance. Electrodes with a higher roughness are expected to exhibit a lower impedance than smoother electrodes due to increased effective surface area and thus a higher capacitance. Related techniques, such as adding porous platinum or roughening gold, are well-known to decrease impedance by orders of magnitude (Prasad et al., 2014; Boehler et al., 2020). We anticipated that the electrodes on embossed LCP, which had a highly rough surface at the microscopic level (i.e., ~2 μm) would show measurably lower impedance than those on pristine smooth LCP. All measurements were performed in physiological phosphate-buffered saline (PBS, pH ~7.4) at room temperature (~22–25 °C). All impedance measurements were performed both before and after accelerated aging treatments, to monitor electrode stability.

### Reactive-accelerated-aging (RAA) soak tests

Long-term electrical reliability can be explored via heated soak tests, which take advantage of the thermal acceleration of the aging processes (Kuliasha and Judy, 2018; Jiracek-Sapieha et al., 2023; Fluker and Judy, 2025; Takmakov et al., 2010, 2015; Caldwell et al., 2020). In this work, fully encapsulated test devices (i.e., those with internal metal traces and both buried and exposed electrode openings) were immersed in PBS at 87 °C with 10 mM hydrogen peroxide, which was added to increase the oxidative stress of the test. This high temperature is expected to significantly speed any possible failure modes (e.g., moisture ingress and material degradation) compared to 37 °C. Following an Arrhenius relationship rule-of-thumb (i.e., the thermal acceleration rate doubles for each 10 °C above the body temperature of 37 °C leads to an acceleration factor of a 2 ^(87°*C*−37°*C*)/10°*C*^ = 2^5^ = 32X). We monitored changes in trace-to-trace isolation resistance and trace resistance over time as indicators of encapsulation failure. Specifically, we measured the resistance of all embedded bonded interdigitated electrodes (IDE) and serpentine test structures at different time intervals to quantify any changes over time. We ran the RAA soak test for 12 days, which is equivalent to about 12 months in the body.

## Results and discussion

### High-resolution metallization on embossed HT-LCP

**Figure 4:**
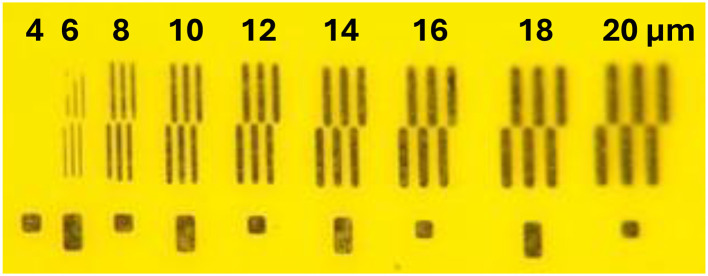
Optical photograph of test structures used to quantify the resolution limits of using lift off to pattern thin sputtered metal films on HT-LCP. The test structures contained metal lines and gaps with nominal line/space dimensions stepping from 4 to 20 μm.

We characterized our ability to pattern thin metal films on embossed HT-LCP coupons using optical lithography, sputtering, and lift off. Using integrated test structures that have sets of parallel metal lines with a width and gap that ranged in size from 4 to 20 μm (Figure 4), we determined that 4-μm-wide features could not be cleanly resolved, 6-μm-wide features showed partial definition, and 8-μm-wide features appeared well defined and reproducible. We also used metal interdigitated electrodes (IDE) of various gaps and serpentine traces of various widths as electrical test structures to assess our ability to reliably pattern narrow lines and gaps using thin metal films on embossed HT-LCP. Measurements of all IDEs revealed that those with a 4-μm-wide gap between metal lines were shorted together (failed) and all IDEs with larger gaps, (i.e., 6, 8, 10, 12, 15, 20, 25, 30 μm) were open circuits (good). Measurements made with the serpentine test structure demonstrated that long 8-μm-wide serpentine traces were reliably continuous while narrower traces (i.e., 4 and 6 μm) were not. As a result, to define the sputtered metal layers on embossed HT-LCP using the nLOF-based lift-off process, we used a minimum design rule of 8 μm line/space. This limitation likely reflects a combination of process-related factors, including the roughness of the embossed surface, resist thickness and lift-off behavior, and non-conformal metal deposition on the micron-scale topography, rather than a fundamental limitation of the LCP material itself. Future work may enable smaller feature sizes through optimization of lithography and deposition processes.

**Figure 5:**
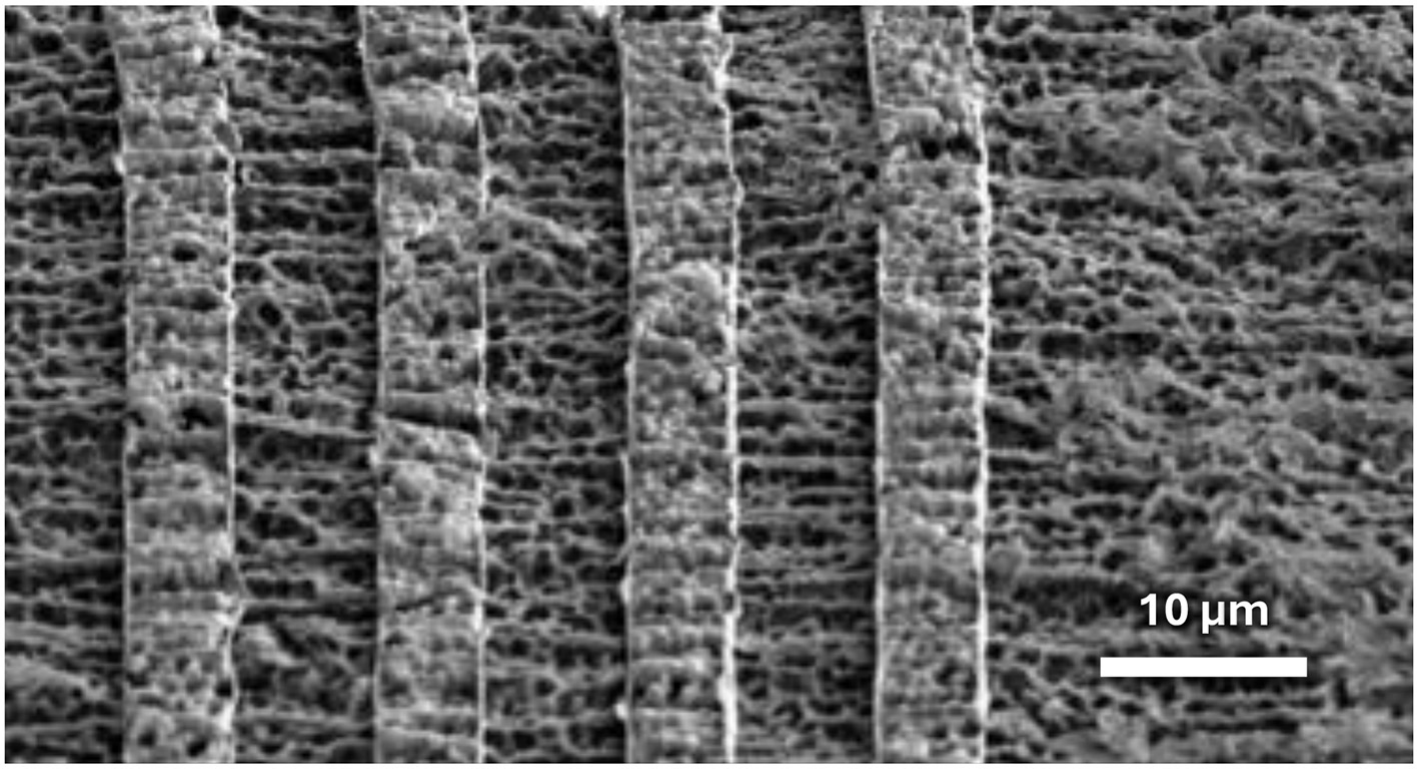
SEM image of 500-nm-thick metal traces patterned on embossed HT-LCP using lift off. The roughness of the embossed LCP is largely maintained in the areas covered with the thin metal layer.

**Figure 6:**
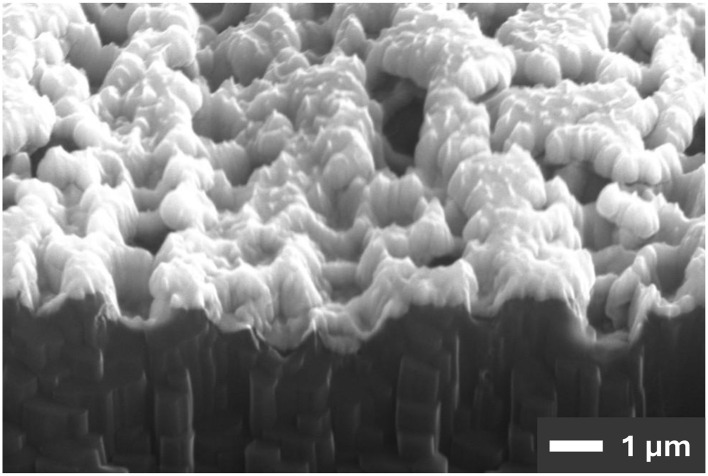
SEM image of a focused-ion-beam cross section of a relatively conform 250-nm-thick layer of sputtered metal deposited on the embossed surface of HT-LCP.

The sputtered metal traces conform to the rough surface of the HT-LCP (Figure 5). Although sputter deposition cannot deposit thin films as conformally as atomic layer deposition, it can achieve much better step coverage than evaporation. Compared to evaporation, sputtering has a much wider source of metal (i.e., 75 mm or larger compared to < 3 mm), operates at a much higher pressures (>5 mtorr vs. < 1 μtorr) and a corresponding shorter mean free paths (~1 cm vs. 50 m), and deposits with enough energy to supports significant surface migration. Although sputtering may not be perfectly conformal, sputtering can deposit a relatively conformal layer of metal on the embossed surface of HT-LCP (Figure 6).

### Effect of metallization on LCP-LCP bonding

One important question was whether an internal metal layer would compromise the mechanical integrity of the LCP-LCP bond. In principle, a metal film could act as a non-bonding spacer, interrupting strong polymer-polymer bonding. To explore this, we directly compared peel strength for bonds formed between LT-LCP and bare HT-LCP vs. LT-LCP and HT-LCP coated by a 500-nm-thick layer of sputtered metal with a gold surface. Each type of test sample was bonded under identical bonding conditions (i.e., bonded for 12 min dwell at 285 °C and 0.25 MPa). Each condition was tested on three independently bonded strips.

**Figure 7:**
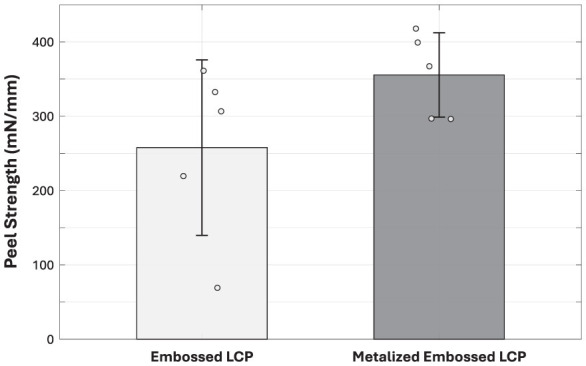
Graph comparing the peel strength of the LT-LCP bonded to embossed HT-LCP to the peel strength of LT-LCP bonded to embossed HT-LCP coated with a 500-nm-thick layer of metal. All bonds were made with the same process conditions (285 °C, 0.25 MPa, 12 min dwell). Error bars indicate one standard deviation. The presence of the thin metal layer does not degrade bond strength and appears to slightly reduce its variability. The small increase in mean peel strength for the metallized samples is within the experimental variability.

As shown in Figure 7, no systematic reduction in peel strength was observed for the metallized interfaces. In fact, the samples coated with the thin metal layer showed less experimental variability than the uncoated samples. Materials failure during testing was predominantly cohesive within an individual layer LCP rather than at the bond interface. Taken together, these results indicate that a sputtered 500-nm-thick layer of metal is effectively “transparent” to the bonding process (i.e., the LCP can still flow into and around the metallized embossed HT-LCP surface). Therefore, fine patterned metal traces do not appear to be a limiting factor for bond integrity under the optimized bonding conditions used in this work. Although the average peel strength of the metallized samples was slightly higher, the standard deviations overlap and the difference is not statistically significant given the small sample size (*n* = 5). One possible explanation for the slightly higher mean value is that the embedded metal layer has a higher elastic modulus than the surrounding polymer and may locally reinforce the mechanically interlocked interface.

### Thinning LCP with RIE

Our experiments demonstrated that at a high level of power (500 W), the etch rate of LCP in RIE and its uniformity depended significantly on etch chemistry. As shown in Table 1, the etch rate of LCP when using an O_2_ + CF_4_ RIE was ~3 times faster than when using an O_2_ RIE. In addition, etching LCP with an O_2_ + CF_4_ RIE was significantly (2–3 times) more uniform than when using an O_2_ RIE. We explored the use of RIE at higher pressure (1,000 mTorr), but the uniformity of the etches was significantly worse, which made it unsuitable for controlled thinning of LCP. We also note that the etch rates for LT-LCP and HT-LCP were very similar.

**Table 1:** Table of RIE etch rate and uniformity for LT-LCP and HT-LCP for different gas compositions and pressures.

Material	LT-LCP	LT-LCP	HT-LCP	HT-LCP
Gases	O_2_ + CF_4_	O_2_	O_2_ + CF_4_	O_2_
Flow rate (sccm)	40/10	40	40/10	40
Pressure (mTorr)	100	100	100	100
RIE power (W)	500	500	500	500
Etch rate (nm/min)	790	330	870	290
Uniformity (%)	90	83	97	87

Overall, low-pressure (100 mTorr) O_2_/CF_4_ plasma conditions provided the best balance between etch rate, uniformity, and preservation of fine metallized features for both LT-LCP and HT-LCP. Under these conditions, thinning could be performed at practical rates while avoiding the poor etch-rate uniformity that limits aggressive thinning at higher pressures. Since the etch-rate non-uniformity was ~10%, reliable LCP thinning of 25-μm-thick LCP sheets by RIE are limited to thicknesses of ~5 μm.

### Reactive-accelerated-aging (RAA) soak tests and electrical reliability

**Figure 8:**
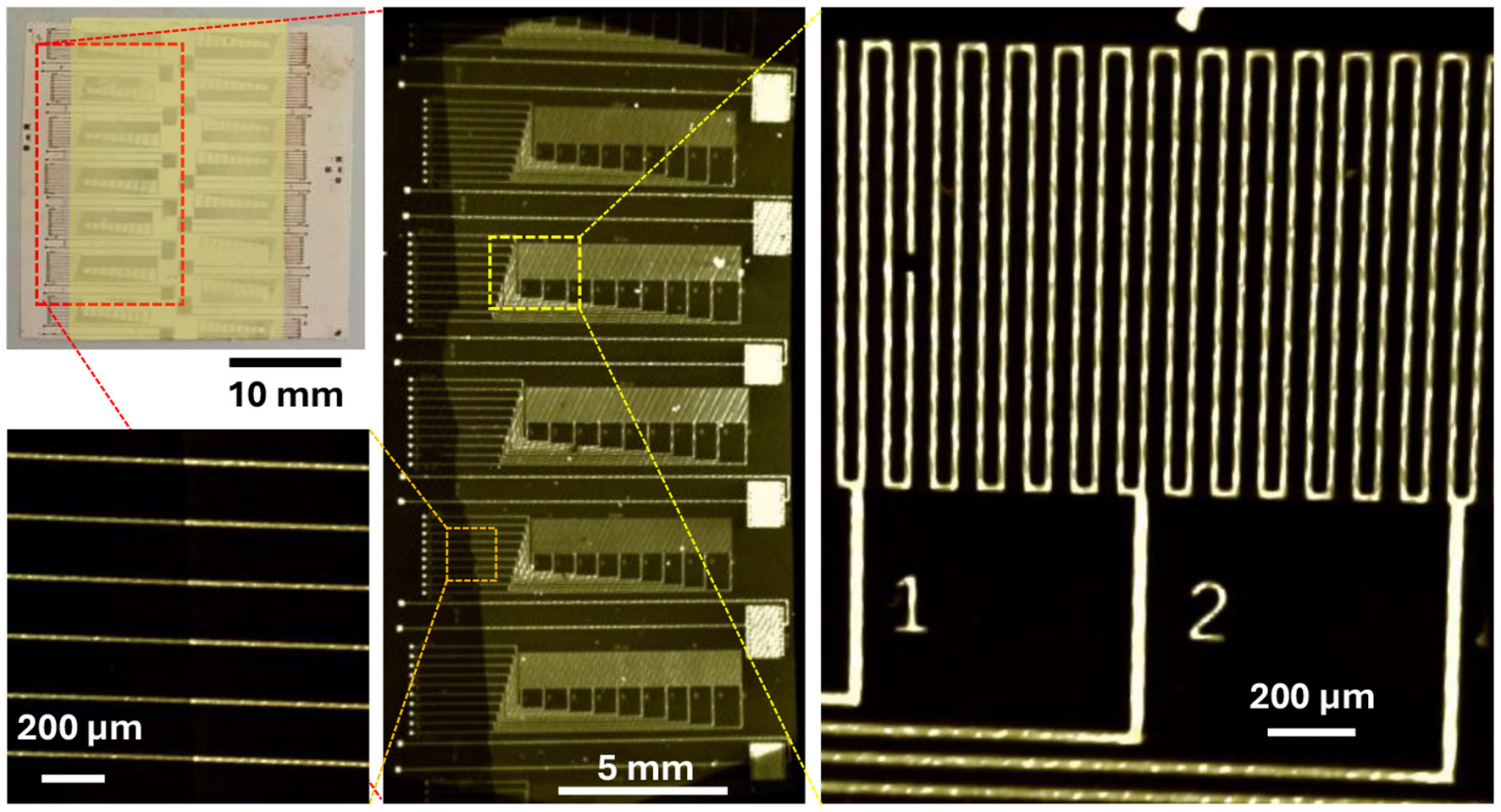
NanoCT images of bonded LCP test structures with integrated patterned metal traces prior to RAA. **(upper left)** Image of the fully bonded HT-LCP/metal/LT-LCP coupon. (center). Image of test structures in the bonded region. **(right)** High-magnification image of the buried serpentine traces. The metal defect occurred during lift off. (lower left) Image of 25 μm **(left half)** outside of the bond area and **(right half)** in the bond area. No line distortion caused by the bond can be observed. Overall, these images demonstrate that well-executed LCP bonding preserves the integrity of fine metallization.

We evaluated the electrical reliability of our devices made with a metal layer that is much thinner than the roughness of the embossed HT-LCP material by making measurements of integrated test structures before bonding, after bonding but before soak testing, and after 12 days of soak testing. The integrated test structures include 200-mm-long metallized serpentine traces with a range of widths and pairs of interdigitated electrodes (IDE) with a range of gaps between the electrodes. Before exposing any samples to RAA, we first confirmed that the bonding process itself did not damage or distort the embedded metal. NanoCT imaging of bonded LT-LCP/metal/HT-LCP coupons showed a sharp interface between the top and bottom LCP layers and clearly resolved the buried thin-film metal traces (Figure 8). Even for the narrowest features, the bonded interface preserved trace integrity (i.e., the lines remained continuous and well-resolved, with no signs of deformation, such as necking or stretching, or mechanical failure such as cracking or breakage). These structural observations indicate that properly tuned high-temperature and high-pressure LCP bonding appears to preserve the geometrical integrity of long and narrow metal lines and does not introduce hidden damage that could compromise subsequent electrical reliability.

**Figure 9:**
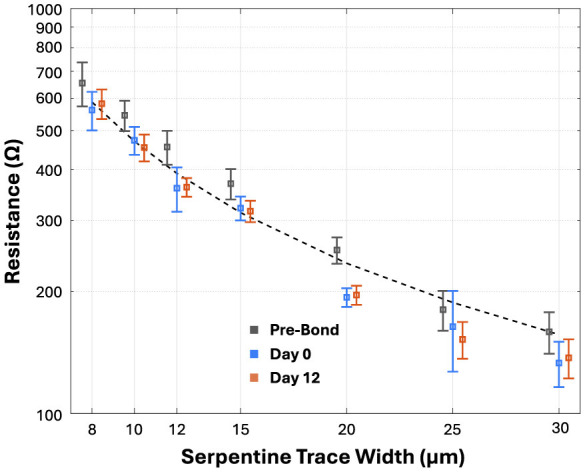
Electrical stability of encapsulated metallized serpentine traces under RAA. LCP-LCP bonded devices containing Au serpentine traces of various widths. Samples were soaked in Reactive Accelerated Aging solution for 12 days (equivalent to ~12 months *in vivo* due to an acceleration factor of 32) and measured periodically. Resistance values of good traces provided before bonding, immediately after bonding (day 0) and before soak testing, and after 12 days of RAA soak testing (boxes indicate the mean and the error bars are for one standard deviation). Each data point represents measurements from multiple trace segments (*n* > 10 depending on trace width and device yield).

We also assessed the impact of the bonding process directly on the electrical properties of the test structures made with the thin metal layer. We did this by measuring the resistance of 20-mm-long serpentine traces between a series of tap points spaced along the 200-mm-long test structure (Figure 9). Since the resistance of a thin-film metal Au trace is given by *R* = ρ*L*/(*wt*), with resistivity of gold ρ, conductor length *L*, trace width *w*, and metal thickness *t*. We expect the resistances of these test structures with ρ ≈ 24 *nΩ*·*m*, *L* ≈ 20 *mm*, *w* ≈ 24 *um* and *t* ≈ 500 *nm*, to be on order of ~40 Ω. The resistance value will scale inversely with width for the different trace widths test (i.e., the resistance of a 4-μm-wide segment is expected to be approximately 6 times higher ~250 Ω). These estimates were based on a relatively low published value for the thin-film resistivity of Au. We did not measure the sheet resistance of our deposited metal. In practice, the measured values shown in Figure 9 are ~5 times higher than our theoretical estimates. The difference in resistance is attributed to four things: (1) the resistivity of the deposited metal may be higher, (2) contact resistance was not included in our estimates, (3) depositing a thin metal film on an embossed surface, which has a roughness several times larger than thickness of the metal, will result in sidewall deposition that is thinner; (4) the conduction path along the embossed surface will be significantly longer due to its tortuosity. Importantly, we observe that there is not a statistically significant difference between the resistances measured before bonding and after bonding. This quantitatively confirms the results of the qualitative NanoCT-enabled visual assessment.

We then subjected bonded devices to 12 days of RAA at 87 °C and a maintained concentration of 10 mM H_2_O_2_, which roughly corresponds to 12 months *in vivo*. Each test structure was periodically assessed by measuring the electrical isolation resistance between the IDEs and we also again measured the conduction resistance of the serpentine traces. Measurements of the IDEs revealed that all with a 4-μm-wide gap were shorted out at Day 0 and all with a larger gap (i.e., 6, 8, 10, 15, 20, 25, and 30 μm) maintained an isolation resistance greater than 0.5 GΩ for all 12 days. In addition, we note that the resistance of the serpentine test structures also did not change after a 12-day-long RAA soak test. In addition to electrical measurements, the bonded devices were visually inspected using optical microscopy before and after the RAA soak test. No visible discoloration, surface erosion, or delamination of the LCP layers was observed after 12 days under these strong oxidative aging conditions. Together, the data from these reliability-focused experiments indicate that LCP-metal-LCP bonding is not only gentle enough for even very thin metal layers to survive the manufacturing process but that the bond is strong and robust enough to survive prolonged exposure to aggressive aging conditions without significant degradation.

### Alignment accuracy when using opaque LCP laminates

**Figure 10:**
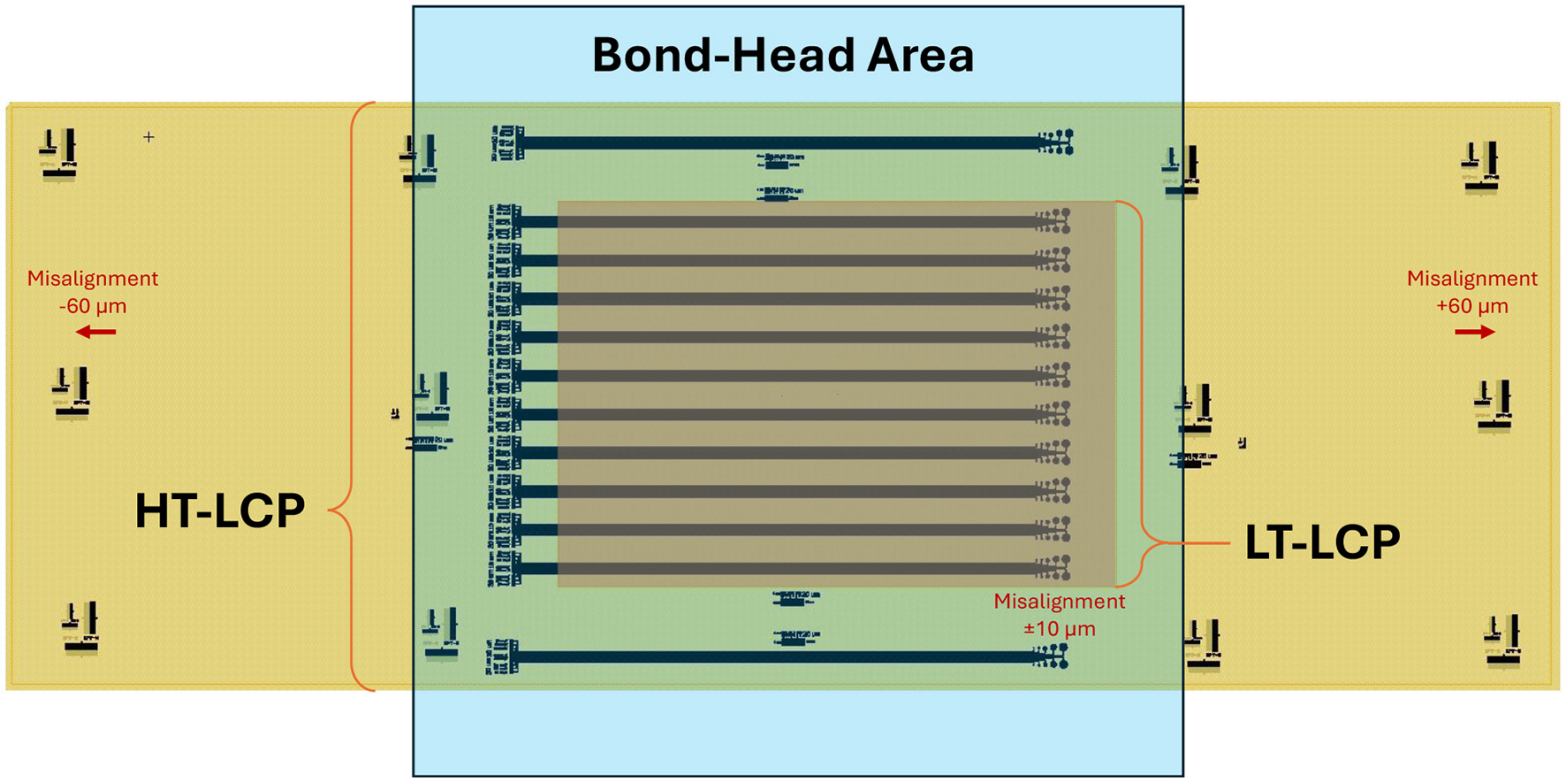
Schematic illustration of etch-mask alignment with bonded LCP-metal-LCP devices. While etch-hole alignment to electrode pads were 10 μm, the outermost structures on the HT-LCP were misaligned to the mask by ~70 μm (in opposite directions on each side). Alignment inside the bonded area remained consistent while misalignment outside the bond area was due to shrinkage of the HT-LCP during the bonding process.

Because the LCP layers were optically opaque both before and after bonding, post-bond alignment of the electrode-punch-through mask could not be performed directly to the buried metal traces. Although the thinning of the entire LT-LCP coupon by dry etching made it somewhat less opaque, direct optical alignment was challenging. Instead, alignment was performed using alignment structures located well outside the bond area on the larger HT-LCP coupon (Figures 3, 10). This alignment method enabled fabrication of aligned electrode openings without requiring direct visibility of the embedded metal layer as described in the Methods and the section above. Although alignment of the etch holes to the electrodes and other features inside the bonded area could be reasonable for larger electrodes (±10 μm), we noticed significant misalignment (~60 μm) in opposite directions on each side of the HT-LCP coupon (Figure 9) outside of the bond area. This pattern indicates that the alignment error is due to a dimensional distortion (i.e., shrinkage and crinkling) of the larger HT-LCP coupon outside of the bonded area. This distortion is likely associated with thermomechanical stresses generated during the bonding process, where the LCP-metal-LCP stack is heated to 285 °C and subsequently cooled, causing differential thermal contraction between the HT-LCP substrate, the thin metal layer, and the LT-LCP encapsulation layer.

Although this thermal expansion mismatch can generate residual stresses in the laminate, the bonding process occurs under pressure in a clamped environment, which helps constrain dimensional changes until the structure cools to lower temperatures (Figure 10). We were concerned that these thermal stresses, combined with the bonding pressure, could potentially damage the thin and narrow metal traces. However, the electrical measurements presented in Figure 9 demonstrate that the resistance of the traces remained unchanged after bonding, indicating that the thin metal layer did not experience significant cracking or electrical degradation during the bonding process. The ability of the metal layer to conform to the micron-scale roughness of the embossed LCP surface may also allow the traces to accommodate small thermomechanical strains without forming continuous cracks. As a result, we were able to use this alignment method to facilitate removal of the LCP above the electrodes and enable interface functionality and the electrochemical characterization of the electrodes.

### Electrode impedance and functional performance

**Figure 11:**
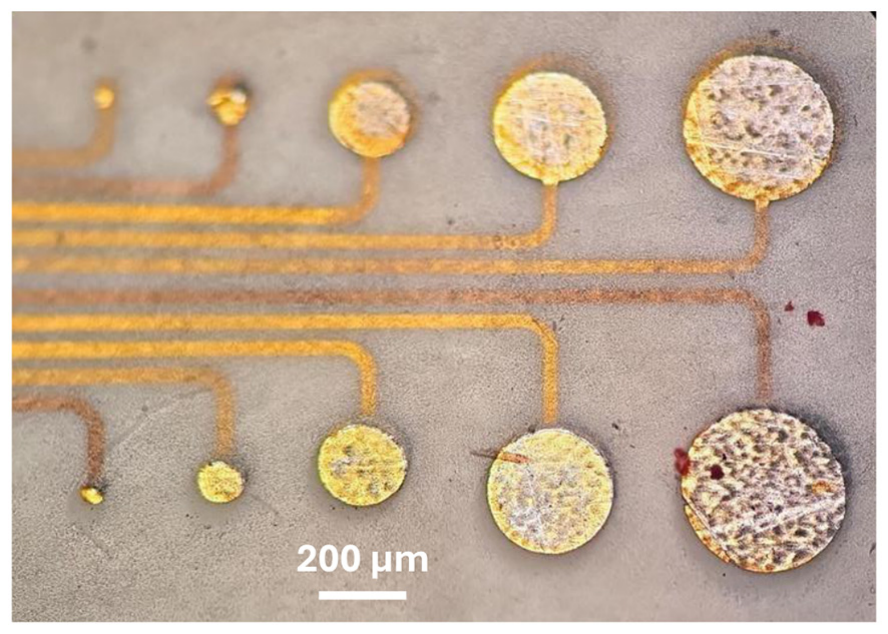
Optical image of a bonded LCP-metal-LCP electrode test structure after thinning the LT-LCP and etching holes through the LT-LCP to the electrodes. Due to the thinning of LCP from 25 μm to 5 μm, the buried traces are now clearly visible. Electrode sizes ranged from 50 to 400 μm.

After aligning the etch-holes mask to the electrode metal layer, the thinned LT-LCP was removed above the electrodes to reveal their metal surface. As shown in Figure 11, the etched circular electrode openings were cleanly formed and centered over the underlying metal pads across the tested range of electrode diameters. This enabled us to use electrochemical impedance spectroscopy (EIS) to characterize the electrodes. Since platinum is a better and more robust electrode material, we then deposited and patterned a 100-nm-thick sputtered Pt layer on top of each electrode prior to the LCP-bonding step.

**Figure 12:**
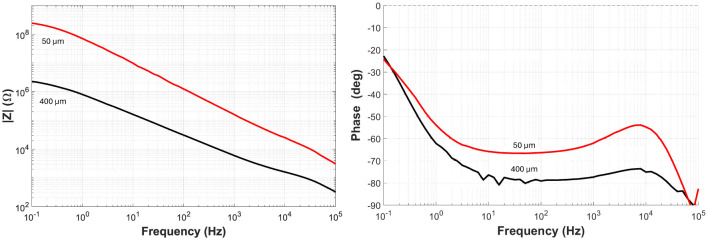
Plots of electrode impedance **(left)** and phase **(right)** as a function of frequency for the smallest (50 μm) and largest (400 μm) diameters tested.

As shown in Figure 12, plots of the magnitude of the impedance vs. frequency decreased as the diameter of the electrode increased. At a frequency of 1 kHz, the impedance of the metallized embossed electrodes with a diameter of 50 and 400 μm was 170 and 6 kΩ, respectively. Compared to experimental results obtained with flat sputtered Pt electrodes, the impedance of the metallized embossed electrodes is ~2X less for the same geometry. This reduction in impedance is linked to the increase in electrode surface area due to their rough surface. These results validate the ability of thin metal traces and electrodes to be reliably integrated onto embossed HT-LCP and bonded to LT-LCP as a new thinner and more robust LCP-based neural-interface technology for recording and stimulation applications.

## Conclusion

We have developed and characterized a fabrication process designed to produce thin and yet robust LCP-based neuroelectronic interfaces. Our process integrates a 500-nm-thick metal layer onto embossed HT-LCP using lift off. Unlike all prior work, which integrates a much thicker and smoother metal layer onto LCP, our thin metal layer conforms to the embossed surface without significantly reducing its roughness. As a result, the thin metal layer supports a much stronger bond to the LT-LCP layer than possible with prior approaches and its presence does not reduce the bonding strength between the LT-LCP and HT-LCP layers. We can reliably produce very long 8-μm-wide lines and spaces that are 4 times thinner than the 2 μm roughness of the embossed HT-LCP that survive the LCP-metal-LCP bonding process without any significant change. Qualitative visual assessments using NanoCT and quantitative assessments using electrical measurements of buried 20-mm-long interdigitated electrodes and 200-mm-long serpentine traces confirm this result. Unlike polyimide-based neural interfaces, a 12-day-long RAA soak test does not negatively impact the electrical properties of the buried traces encapsulated within the bonded LCP layers. An RIE-based thinning process can rapidly reduce the thickness of the 25-μm-thick LCP layers to 5 μm with >90% uniformity. Due to the roughness of the exposed metal surface, the impedance of the electrodes is 2 times lower than equivalent flat electrodes. As a result, we have demonstrated that it should be possible to use this process technology to produce much thinner LCP-based neural interfaces that have tremendous robustness, reliability, thinness, and flexibility.

## Data Availability

The raw data supporting the conclusions of this article will be made available by the authors, without undue reservation.
